# Combining portable coagulometers with the Internet: A new model of warfarin anticoagulation in patients following mechanical heart valve replacement

**DOI:** 10.3389/fsurg.2022.1016278

**Published:** 2022-10-12

**Authors:** Yu Huang, Lei Huang, Zhen Han

**Affiliations:** Department of Cardiovascular Surgery, Peking University Shenzhen Hospital, Shenzhen, China

**Keywords:** mechanical valve replacement, Internet, warfarin, self-management, review

## Abstract

Heart valve replacement, as a safe and effective treatment for severe valvular heart disease, can significantly improve hemodynamics in patients. However, such patients then require lifelong anticoagulant therapy. Warfarin, a cheap and highly effective vitamin K antagonist, remains the major anticoagulant recommended for lifelong use following mechanical heart valve replacement. However, the effect of warfarin anticoagulant therapy is complicated by physiological differences among patients and non-compliance with treatment at different degrees. Effective management of warfarin therapy after heart valve replacement is currently an important issue. Portable coagulometers and the emergence of the Internet have provided new opportunities for long-term management of anticoagulation therapy, but the safety and affordability of this approach remain to be fully evaluated. This paper reviews recent progress on the use of portable coagulometers and the Internet in the management of warfarin anticoagulation therapy following mechanical heart valve replacement, which offers opportunities for reducing complications during postoperative anticoagulation and for facilitating patient compliance during follow-up.

## Introduction

Valvular heart disease, as the main cause of heart function disorder, reduces quality of life and can be fatal. Epidemiological surveys show that the incidence of valvular heart disease is gradually increasing as the global population ages (1–3), and it has attracted heightened concern in the practice of cardiac surgery and medicine. Unlike other major cardiovascular diseases such as atrial fibrillation, valvular disease as an organic disease of the heart lacks effective drug therapy. Since the beginning of this century, interventional therapy has developed rapidly and demonstrated potential advantages for treatment of some high-risk valvular diseases. However, for patients with valvular diseases for whom intervention is indicated, open-heart surgery is still the standard approach.

Heart valve replacement, as a safe and effective clinical treatment, can significantly improve cardiac hemodynamics and quality of life (4). In terms of the choice of replacement valve, various options exist because of the different material properties of mechanical biological valves. Studies have shown that the risk of bleeding is about one-third less in patients with a biological valve than with a mechanical valve, although the risk of repeat surgery is more than three times higher (4, 5). Therefore, the current consensus is that mechanical valves are more suitable for younger patients. Interaction between artificial materials and blood can easily lead to thrombosis on the valve surface, which can cause stroke and even death in severe cases. Clinical guidelines recommend that patients undergoing mechanical heart valve replacement take anticoagulant drugs for life (5–8).

Warfarin is a highly effective and inexpensive vitamin K antagonist that is widely used for preventing blood clot formation in artificial joint replacements, venous thromboembolism, atrial fibrillation and heart valve surgery. However, the therapeutic window for warfarin is narrow, and significant differences exist among individuals in its effect on blood coagulation. Patient genotype, gender, age, education, and compliance with clinical follow-up can affect the warfarin efficacy to varying degrees (9, 10). Studies have shown that increasing the frequency of anticoagulant index measurements is helpful for improving the efficacy of oral anticoagulants and reducing the occurrence of bleeding, thromboembolism, and other complications (11, 12). Traditional management of anticoagulant outpatient services is cumbersome. Patients have to go through outpatient registration and have blood drawn for a hospital or off-site laboratory to obtain coagulation indices, such as the international normalized ratio (INR). Finally, the outpatient doctor or pharmacist determines an INR target value and prescribes a dose adjustment plan. With these complicated procedures and long patient-waiting times, regular patient monitoring has become challenging.

Development of point-of-care (POC) INR determination by bedside real-time portable coagulometers has revolutionized management of warfarin anticoagulant therapy, allowing patients to self-test and self-manage their anticoagulant therapy conveniently. In recent years, the popularization and rapid development of the Internet have provided new opportunities for clinical anticoagulation monitoring and post-discharge follow-up (12–14). In this review, we summarize the latest research on warfarin anticoagulant therapy that takes advantage of new developments in Internet technology and self-testing. This review has three sections. First, we review management of anticoagulation-based on fixed network terminals. Second, we discuss progress in anticoagulation management based on mobile terminals and focus on several large clinical trials conducted in China. Finally, we analyze problems that stand in the way of further development of portable coagulometers connected to the Internet for managing anticoagulation therapy after cardiac valvular disease. Our discussion is intended to provide inspiration for improved postoperative follow-up management and to reduce anticoagulation complications.

## Summary of the warfarin anticoagulant self-management model

Point-of-care testing (POCT) allows for testing physiological specimens, such as fingertip (venous) blood, collected using portable analytical instruments to facilitate access to results in a short period of time. Currently, the INR is the main detection index describing anticoagulation. Mainstream anticoagulant instruments are based on electrochemical or physical principles (including optics, acoustics, and electrical properties) and make use of a paper test card (15). Currently, instrument manufacturers, such as Roche, have made portable coagulometers commercially available on a large scale. Compared to the large instruments used in hospitals and laboratories for testing blood, portable coagulometers are easy to operate, require less time and space, allow for more frequent testing, and significantly improve patient quality of life.

### Measurements of blood coagulation based on electrochemical principles

An electrode and a dry reagent are placed on a test card in advance. Upon interaction between the blood and paper, oxidation of thrombin by the substance in the reagent occurs, generating an electric current. The INR of the blood sample is calculated based on changes in current (16, 17).

### Measurements based on optical principles

Coagulation changes the optical properties of blood, and the INR of coagulation is calculated by measuring its viscoelasticity and refractive index. Therefore, instruments contain a light source and optical detector. For Cascade POC (Helena Laboratories Point of Care, USA), the test card contains magnetic iron oxide particles and other reagents. An electromagnetic field causes the iron oxide particles in the reagents to move, which causes fluctuations in the optical transmittance of the fluid. Blood entering the card triggers coagulation process, which reduces movement of the iron particles and thereby stabilizes the transmittance signal, upon which the instrument calculates the corresponding INR (18).

### Measurements based on acoustic principles and optical detection

The approach is similar to that described above. Blood coagulation changes the basic acoustic state of the liquid, and by observing changes in the resonant frequency of the blood, changes in its viscosity can be analyzed and the time course of coagulation time can be obtained. Effective acoustic measurement devices include quartz crystal mass (QCM) balance resonators and surface acoustic waves (19). A portable coagulation detection method based on a QCM sensor has been proposed that allows researchers to obtain dissipative factor results and coagulation indicators *via* smartphone analysis. And it was proved that the test was as accurate as conventional methods based on large machines (19).

### Measurements based on electrical principles

The resistance of a liquid is closely related to its composition. Clotting is a complex physiological process. A series of changes in clotting factors eventually lead to fibrin aggregation and coagulation, thus changing the electrical resistance of blood. Coagulation can be monitored indirectly by detecting the rate of change of blood resistance over time. A microfluidic test platform with screen-printed electrodes and a fully integrated single-chip impedance analyzer has been developed by Chen (20). In this design, constant frequency voltage excitation is provided, and the differential impedance signal is recorded to calculate the coagulation time.

These different design principles do not lead to large differences in the effectiveness of clinical testing. Patients and medical staff can choose according to clinical needs, economic resources, and requirements of medical insurance policies. Current mainstream portable clotting devices include CoaguChekXS (Roche), ProTime (ITC), INRatio (Hemosense), and Harmony (Johnson / Johnson) (21–26) (Table 1).

**Table 1:** Characteristics of point-of-care INR devices licensed for use in Canada*.

Device	CoaguChek S Roche Diagnostics GMBH	CoaguChek XS Roche Diagnostics GMBH	CoaguChek XS Plus Roche Diagnostics GMBH	ProTime 3 International Technidyne Corp	INRatio Hemosense Inc.
Target group	Patient or professional use	Patient or professional use	Professional use only	Patient or professional use	Patient or professional use
Approximate cost of monitor (USD)	n/a	499	1,499	1,800	600
Approximate cost of test strips/cuvettes (USD)	6 test strips: 50.25 24 the test strips: 200.88	6 test strips: 50.25 24 the test strips: 200.88	6 test strips: 50.25 24 the test strips: 200.88	Box of 25 cuvettes: The 145.00	12 test strips: 80.00 48 test strips: 298.00
Blood sample	10 μl of whole blood (venous or capillary)	10 μl of whole blood (venous or capillary)	10 μl of whole blood (venous or capillary)	27 μl of whole blood (venous or capillary)	15 μl of whole blood (capillary only)
Detection principle	Iron oxide	Electrochemical	Electrochemical	Optical clot detection	Electrochemical
Memory store	60 tests with time and data	100 tests with time and date	500 tests with patient details, time, and date	30 tests with time, date, and quality control results	60 tests with time, date, and quality control results
Quality control	Liquid quality control	Strip integrity check	Liquid, strip integrity check	Internal (2 levels) and liquid	Internal (2 levels) only

* Data from references (21–26).

The main attraction of portable clotting machines is that they require less time to test the blood when compared with traditional testing methods that involve large machines. Studies have shown that hospital testing laboratory requires 65.02 ± 24.5 min to test INR, whereas portable coagulation tests require only about 1–4 min (27, 28). The design principles of mainstream portable blood coagulation instruments do not differ greatly, and product upgrades focus mainly on improving convenience and user comfort.

“Self-management” in patients receiving warfarin anticoagulant therapy refers to patients monitoring their INR at home using a portable coagulometer and adjusting the dose of warfarin by consulting treatment guidelines or a clinician. Studies have shown that self-management of anticoagulant therapy in the early postoperative period can improve initiation of medication and detection of blood clotting, maintain INR in a lower range, reduce the occurrence of complications such as severe bleeding and thromboembolism, and have significant pharmacoeconomic advantages (29, 30). Gallagher et al. (31) reported that when patients switched to INR self-monitoring and online management, short-term costs were significantly higher than those of outpatient anticoagulation management, with a difference of about €60 between the two groups over a 6-month period. This is mainly because current devices are expensive and health insurance policies do not completely cover the costs.

Most researchers believe that the use of Internet technology combined with self-testing INR can reduce adverse events in anticoagulation therapy. If warfarin-related adverse events are included in the medical costs, the new management model may be more economical in the long run, especially for patients whose homes are far from the clinic (32–34). One study (35) revealed that, compared with routine care, self-monitoring saved 1,187 GBP per person over the past 10 years. More than 112 million GBP could be saved if 10% of the current 950,000 patients switched to portable devices within 10 years.

In the following section, we primarily focus on how portable blood coagulation instruments can be effectively used with the Internet and different terminals for economical and effective warfarin therapy following heart valve replacement.

## “Internet +” applied to warfarin anticoagulation management in patients after heart valve replacement

### “Internet+Medical”

**Figure 1:**
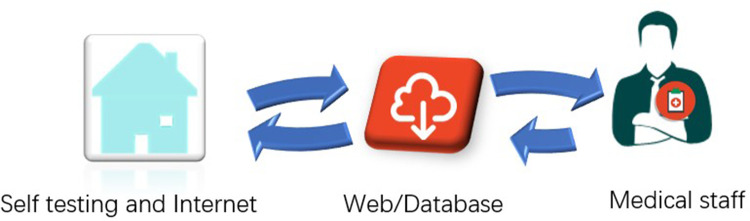
Model of warfarin anticoagulation therapy combining Internet and INR self-testing.

“Internet + medical” refers to the application of Internet technology to traditional medical fields. Researchers initially attempted to integrate the Internet with the medical field to expand education on diseases, diagnosis, and treatment, and to outpatient follow-ups. This application did not only provide a reliable and convenient means for individuals to obtain information about health and disease control and prevention but also for patients to access help from medical institutions (36, 37). It also improved clinical practice by making it more convenient and personalized. At present, the “Internet + medical” model is basically stable, including doctor-patient entities and online diagnosis and treatment systems and databases. Doctor-patient entities include doctors, nurses, pharmacies, clinics and patients. Online diagnosis and treatment systems mainly include online registration, consultation systems, electronic medical records and settlement and evaluation systems. Databases are generally individual or public. Individual databases contain all tests, examination results and medical records of patients, and are only accessible to doctors, nurses and patients to facilitate diagnosis and treatment. Public databases contain anonymized patient information that has often been processed in various ways and which allow limited access to medical personnel, researchers, patients, social enterprises and government regulatory agencies. Therefore, Internet-based anticoagulation management should adhere to the requirements of offline entities and online procedures, as shown in Figure 1.

## Computer-based fixed communication and self-management of anticoagulation

Computer-based fixed communication refers to that in which customers connect to computers or network equipment mainly through fixed lines, such as cables and optical cables. It is characterized by fixed connection lines and immobility or limited mobility of network terminals. Therefore, this mode mainly existed in the early development of the Internet and currently in underdeveloped countries or regions. With the development of mobile Internet and artificial intelligence, the drawbacks of this model have become apparent. The invention of the portable coagulometer allowed patients to monitor INR at home and self-administer warfarin according to the test results, which addressed the problem of outpatient anticoagulation therapy. Initial attempts by researchers to have patients test their own INR and consult their doctors on medications resulted in significantly improved patient satisfaction (38–41). However, telephone communication is not always timely; it is difficult to analyze statistically and has other disadvantages. With the popularity of personal computers in foreign countries, clinicians began to try to follow up patients by e-mail, which to a certain extent solved the difficult problem of preservation of patient data. Due to the difficulty in performing professional data analysis by e-mail, researchers later set up professional anticoagulation management websites for guiding patient self-testing of INR at home. This hybrid approach has become a new direction in research on management of anticoagulation therapy, and a number of remote home anticoagulation management systems have emerged.

Finkelstein et al. (42) reported in 2003 that a home automated telemanagement (HAT) system provided multidisciplinary support for patients receiving anticoagulant therapy and improved patient compliance to treatment plan. The system consists of a home unit, a HAT server, and a clinician unit. Patients connect to a coagulation-time detector using a home computer, and their symptoms and test results are automatically sent to the server and analyzed by the system. This was an early demonstration of an Internet-based medical expert system. This patient-server-physician system design was later incorporated into remote anticoagulation management systems. Koertke et al. (43) analyzed data from 1,571 patients who received mechanical valves from January 2006 to April 2012; 1,304 patients underwent aortic valve replacement, whereas 189 and 78 patients underwent mitral valve replacement and double valve replacement, respectively. The patients were randomly assigned to one of three groups: low dose INR self-management (LOW), very low-dose (VLO)INR once, or twice (VLT) weekly testing. Over a period of 2 years, the proportions of patients in the LOW, VLO, and VLT groups without bleeding were 96.3%, 98.6%, and 99.1%, respectively (*P* = 0.008). The corresponding values for thrombotic events were 99.0%, 99.8%, and 98.9%. This study suggests that remotely managed ultra-low-dose INR self-management is comparable to low-dose INR in terms of thrombosis risk and superior in terms of controlling bleeding risk.

We analyzed time in therapeutic range (TTR) outcomes from eight major studies and found that INR self-testing combined with online management could improve TTR (31, 44–51) (Table 2). Additionally, effective patient education has influenced warfarin treatment of valvular heart disease (52). In addition to designing the doctor-patient communication website, other researchers (53) have developed Internet courses to help educate patients about the importance of adhering to a medication regimen, thereby improving their health and reducing thromboembolic events and hospitalizations. With the rapid development of mobile Internet in this century, online management based on websites and e-mail has gradually turned to mobile phones and other mobile terminals, providing additional options for remote warfarin management.

**Table 2:** TTR value comparison using warfarin between online and hospital management*.

Research	Intervention group		Control group		*P* value
	Mean	SD	Mean	SD	
Ryan et al. (31, 44)	72	19.7	59	13.5	<0.05
Verret et al. (45)	80	13.5	75.5	24.7	0.79
Soliman et al. (46)	72.9	11	53.9	14	0.01
David et al. (47)	66.2	14.2	62.4	17.1	<0.001
Khan et al. (48)	71.1	14.5	63.2	25.9	>0.05
Brasen et al. (49)	82.7	12.0	81.6	15.8	0.31
Cao et al. (50)	74.0	30.8	78.9	17.7	0.393
Cao et al. (51)	79.35	26.31	52.38	12.67	<0.001

* Data from references (31, 44–51).

## Mobile Internet and anticoagulant self-managing

Mobile Internet technology removes the requirement that a user's physical connection to the network should be at a specific location (54, 55). The widespread use of smartphones and tablets has allowed online voice calls, text messaging, and third-party apps (APPs) to profoundly change lifestyles. In 2021, the number of internet users worldwide was 4.9 billion, up from 4.6 billion in the previous year (56). Substantial evidence suggested that voice contact and SMS interventions can improve cardiovascular preventive care in developed countries by monitoring factors, such as patients' weight, smoking status, and physical activity (53–55). Over the past decade, new social media sites have been developed, providing opportunities for doctors and patients to interact virtually and share written information, graphic displays, videos, photos, and other forms of communication (57). At present, several patients and medical personnel are able to access and exchange health and medical information through mobile networks. This form of communication provides an opportunity to disseminate health guidance at low cost and may increase the cost-effectiveness of health interventions. Additionally, it can promote social support and influence health behavior (58, 59).

Therefore, the mobile Internet provides more options for anticoagulation management. Patients can access applications through mobile phones, tablets and other terminals to manage drug dose, which can not only improve the frequency of anticoagulation monitoring, but also enable patients to arrange more rapid and efficient return visits without increasing treatment risk, save time and economic cost, and improve quality of life and treatment satisfaction.

Yamamura et al. (60) used a drug therapy support system, CoaguChek, to test INRs, and patients could upload the data to the system at any time using their mobile phones. In the event of abnormal data, the attending clinician conferred with the pharmacist to determine a new warfarin dose and inform the patient *via* e-mail. This initiative was a significant improvement over fixed communications because mobile terminals remove spatial limitations on communication between patients and medical staff. Further, a novel Anticlot Assistant smartphone application has been developed by Li (61), which will provide valuable data for related research. Zhu (62) conducted a prospective, multicenter, randomized, open-label, controlled clinical trial with 1-year follow-up that incorporated a mobile medical network application. Their APP was divided mainly into doctor and patient modules. The doctor could contact the patient in real-time, answer the patient's questions, educate the patient, and guide treatment. As in most studies, the Internet-based group showed a significantly higher TTR, and anticoagulation complications, including bleeding and embolic events, were less frequent in the Internet-based group than in the conventional group (6.94% vs. 12.74%; *P* = 0.01). Jiang et al. (63) developed an APP named “Yixing”, which was not connected to a portable clotting instrument. Compared with follow-up using telephone remote management, this APP established a communication platform for patients and pharmacists, facilitating timely management of warfarin, improving efficiency, and increasing fraction of TTR (FTTR). However, not all studies have reported advantages of the new anticoagulation management model (33, 47).

If significant benefits in terms of increasing TTR or reducing hospitalization and mortality rates cannot be demonstrated, then anticoagulation after heart valve surgery will continue to be a problem for clinicians (64). In a recent study of 383 patients who underwent mechanical heart valve surgery, no significant differences in TTR existed between self-testing combined with the Internet and clinical anticoagulation management (65), which is in contrary to findings from most previous studies. In another recent study (51), the incidence of extreme INR values was significantly lower in patients treated *via* the specialized anticoagulant software “Alfalfa” than in those treated using conventional warfarin management. However, compared with the control management group, patients using Alfalfa had a higher incidence of minor bleeding events, which was attributed to the opportunity for users to more frequently report treatment complications. Patients who are routinely followed up are often questioned over a longer period of time; therefore, they may forget about minor adverse events. Therefore, large-scale and long-term studies are still needed to investigate whether the anticoagulation management model based on self-testing and Internet connectivity is really superior to traditional outpatient management (Table 3).

**Table 3:** Comparisons of adverse events using warfarin under online vs. hospital management*.

Adverse events		Major bleeding events	Minor bleeding events	Total bleeding	Thromboem-bolic events	Hospitalization	Mortality
Ryan et al. (31, 44)	Intervention group	-	-	0	2	-	-
	Control group	-	-	1	1	-	-
Verret et al. (45)	Intervention group	2	24	26	0	9	0
	Control group	1	22	23	0	6	0
Soliman et al. (46)	Intervention group	1	-	1	0	-	1
	Control group	1	-	1	1	-	1
Cao et al. (50)	Intervention group	1	8	9	0	1	-
	Control group	0	7	7	0	0	-
Cao et al. (51)	Intervention group	2	45	47	1	1	-
	Control group	12	20	32	1	12	-

* Data from references (31, 44–46, 49, 51).

Chinese researchers have used QQ social media software to follow up patients undergoing mechanical heart valve replacement. The results show that social media software can yield good follow-up results, and the advantages of QQ communication are greater for patients living in rural or remote areas (66). Chen et al. (67) conducted a non-randomized prospective controlled clinical study. One hundred patients were divided into groups undergoing cardiac valve replacement (40 cases), self-monitoring (40 cases), and self-management (20 cases). A portable blood clot device (CoaguChek Xs) was used for INR self-monitoring, and WeChat instant messaging software was used for communications during follow-up. The TTRs of the three groups were 45.9%, 61.2%, and 65.4%, respectively, and the FTTRs were 48.3%, 60.7%, and 64.9%. The instant messaging software platforms, “WeChat” and “WhatsApp”, have a large number of users and have affected traditional doctor-patient communications by facilitating the safety of patients' anticoagulation treatments, and to some extent alleviating the burden on medical personnel. There is still a lack of large-scale research on anticoagulation management that combines portable coagulometers with social media around the world. Instant messaging tools and social media place low demands on the educational level of patients, but cannot provide for professional statistical analysis of clinical data. Given the limitations of using WeChat, WhatsApp, Facebook, and other social media software to perform anticoagulation follow-up management and help educate patients and their families about cardiac surgery, development of professional anticoagulation management procedures seems inevitable (68–70).

## Problems and limitations

### Both professionalism and universality should be emphasized

Common social media software has a wide audience but lacks medical expertise. It is only convenient for communication between doctors and patients but cannot individualize medical treatment. Additionally, a complex, overly specialized medical device or application can limit patient compliance. Therefore, design of portable coagulation instruments and anticoagulation management procedures should follow the principles of simplicity, convenience and ease of learning. The majority of patients undergoing heart valve replacement are elderly, who have low learning ability and are slow to absorb new information. Complex and cumbersome detectors or applications will reduce the number of eligible patients and reliability of the resulting statistical data.

### Medical devices and clinical models should always be patient-centered

The rights of patients should always be a primary consideration. Consideration of ethical issues in emerging models can help ensure that doctors provide medical services more safely (71). Although self-management of warfarin anticoagulation therapy can significantly reduce monetary cost and enhance clinical benefits, complications, such as bleeding and thrombosis, should not be ignored. Whether using a clotting device or accessing Internet software, medical staff and product designers must consider the timely, effective and appropriate treatment to patients with clinical complications or extreme events. There is no doubt that such intellectual and legal questions are of great importance.

### Cost considerations cannot be ignored

The market price of a Xprecia Stride Coagulation Meter (Siemens Healthcare) is approximately US$ 1428, and the Roche CoaguChek XS Pro Meter costs up to US$ 2,14. As a result, portable clotting machines have been slow to achieve wide use because of their high cost. Moreover, professional anticoagulation management applications are costly to develop and slow to produce a monetary return. Therefore, the relevant studies based on POCT coagulation detectors are only conducted in some large heart centers or hospitals, and studies on their connection with the Internet or mobile terminals, such as smart phones, are rarely reported. There is reason to believe that there will be many such studies during the “Internet plus healthcare” boom, including attempts to include the cost of portable clotting tests in health insurance.

### Cyber security

When patients and doctors use the “Internet +” anticoagulation management model, protecting patients' medical privacy is an essential consideration. As a general rule, patient medical records should not be made known to anyone other than the patient and the attending physician, which is basic privacy protection. However, anonymized patient medical data can be aggregated into public research databases. When patient data is used for medical or policy research, patients should be informed and their data handled according to their wishes. Finally, some national security data must be protected from leaks and attacks.

### Test times and frequency of doctor-patient communication

Although multiple tests can effectively monitor blood coagulation, excessive coagulation testing can undoubtedly increase the time, effort and cost for patients with stable INR levels. Being able to connect online at any time and place can also add to doctors' workload outside of their regular hours. Approaches to achieving a favorable balance between reducing the burden on medical staff and ensuring safety and low cost for patients merits further research.

## What can we expect?

Warfarin, as the most common anticoagulant, is widely used in clinical practice. The rapid development of new technology and ideas has resulted in increased opportunities for warfarin anticoagulation therapy.

To ensure accurate results, next generation coagulation detectors will need to further reduce costs and use new materials and production processes. Related products will need to be more convenient and accurate. Presently, some portable coagulation instruments have Bluetooth, networking, and other functions that allow automatic uploading of test results and alerts in the event of a critical test value. Therefore, future efforts are expected to focus on approaches to ensuring the accuracy of test results and therefore patient safety, and may even lead to non-invasive methods for detection of clotting indicators and artificial intelligence to provide medical care (72).

The Internet has been widely used in the field of medicine. At present, it focuses on two general directions: discovering potential medical clues and guiding personalized treatment. Patients test themselves and upload their INR data, and doctors view patient anticoagulation records at different times and places to comprehensively evaluate patient health status. By careful analysis and evaluation, individual differences in the effects of warfarin and lifestyle, food and other events on anticoagulation stability can be assessed. Widespread adoption of the Internet and self-test INR anticoagulation management model, along with constantly improved databases, can lead to vast amounts of data. This will include warfarin anticoagulation data from different countries and nationalities and ultimately necessitate cloud computing and big data analysis to guide clinical practice.

The use of properly equipped medical apps and tools on smartphones and other mobile devices can reduce the need for specialist care, resulting in significant cost savings for the healthcare system, and “on demand” communication will further promote a harmonious doctor-patient relationship. At the same time, it should be possible to develop a portable blood detector by integrating coagulation index detection with other blood indicators such as blood sugar and biochemical indicators. The anti-coagulation management terminal based on the “Internet +” concept has developed into a chronic disease management platform, which is the direction of future research. In some developing countries, patients in remote areas with large rural populations cannot enjoy the convenience of anticoagulation services in large central hospitals. The Internet + self-monitoring of anticoagulation model has become even more urgent during the COVID-19 pandemic as city closures and population quarantines have resulted in the failure of many patients to receive clinical anticoagulation therapy after mechanical valve replacement, thus increasing the risk of thromboembolic complications such as bleeding. In conclusion, this new model of anticoagulation management is worth pursuing, and there is good reason to expect that it will bring benefits to heart valve replacement patients, clinicians, and the community healthcare system as a whole.

## Conclusion

INR self-monitoring based on portable coagulation instruments and doctor-patient communication based on Internet technology offer many clinical and economic advantages over traditional anticoagulation management schemes for patients following valve replacement. This approach is especially valuable in areas with underdeveloped transportation systems where outpatient visits can be challenging. Legal and cost issues relevant to its development should be considered. Therefore, prospective, multi-center, large-sample clinical studies are warranted to validate this model.
